# Levels and correlates of knowledge of teething among Saudi Arabian families

**DOI:** 10.7717/peerj.13948

**Published:** 2022-08-18

**Authors:** Dina A. Alkhozaim, Sanaa N. Al-Haj Ali, Ra’fat I. Farah

**Affiliations:** 1 College of Dentistry, Qassim University, Almulayda, Qassim, Saudi Arabia; 2 Department of Orthodontics and Pediatric Dentistry, College of Dentistry, Qassim University, Almulayda, Qassim, Saudi Arabia; 3 Department of Prosthetic Dental Sciences, College of Dentistry, Qassim University, Almulayda, Qassim, Saudi Arabia

**Keywords:** False beliefs, Knowledge, Practices, Teething, Saudi parents

## Abstract

**Background:**

Parental knowledge of teething has been repeatedly investigated; however, little emphasis has been made on the associated sociodemographic factors with good knowledge of the parents and whether or not good knowledge is translated into adopting proper pain-relieving practices. The present study aims to firstly assess the knowledge level and practices of Saudi parents regarding teething and then explore associated sociodemographic variables with good knowledge of teething to determine the relation between parents’ knowledge of teething and their adoption of pain-relieving practices.

**Methods:**

This cross-sectional study recruited parents from the social networking sites Facebook, Twitter, Instagram, and WhatsApp, and they were asked to answer a pretested three-part internationally accepted questionnaire. Data were examined using descriptive statistics, chi-square analysis, multivariate logistic regression analysis, and Spearman rank-order correlation coefficient analysis.

**Results:**

One thousand four hundred ninety-nine parents responded and returned completed questionnaires. Of those, only 11.2% had good knowledge of teething. The majority of parents did not have basic knowledge of the teething period as well as believed that teething was associated with a runny nose (87.5%), diarrhea (77.9%), and sleep disturbance (72%). The results of multivariate logistic regression analysis indicated that parents with no primary school education (Odds Ratio: 0.29), or those who had an intermediate or secondary education level (OR: 0.55 and 0.78) were less likely to have good knowledge compared with parents who had a university degree. However, parents who earned intermediate monthly income (OR: 6.63) were more likely to have good knowledge of teething. With regards to practices used to relieve teething pain, inappropriate practices were observed regarding bottle feeding at night to soothe the child’s pain (72%) and applying topical analgesics to rub the gum (72.4%). A significant positive correlation was found between the knowledge score and the practice score of both fathers and mothers (*r* = 0.22 and 0.13, *p* < 0.0001).

**Conclusion:**

A very low percentage of Saudi parents, mainly those with the highest education level and intermediate monthly income, had good knowledge of teething, which translated into appropriate practices to soothe the child’s pain regardless of the parent’s gender. Saudi parents should receive anticipatory guidance related to teething from all health professionals to ensure an uneventful teething period for their children.

## Introduction

Teething, or tooth eruption, is the normal physiologic process when a tooth emerges into the oral cavity from the pre-eruptive location in the alveolar bone (Thompson & Huntington, 2019). It is a universal and recurrent process that is experienced by all children (Wake, Hesketh & Allen, 1999). It commences at 6 months of age and completes when children reach the age of 3 (Elbur et al., 2015; Bankole & Lawal, 2017), and is generally accompanied by symptoms (Macknin et al., 2000; McIntyre & McIntyre, 2002; Wake & Hesketh, 2002; Owais, Zawaideh & Al-Batayneh, 2010).

Massignan et al. (2016) in their meta-analysis indicated that the overall prevalence of teething symptoms is as high as 70.5%. Suggested symptoms included general irritability, disrupted sleep, vomiting, gingival irritation, increased salivation, loss of appetite, diarrhea, circumoral rash, intraoral ulcers, fever, a desire to bite, gum-rubbing, sucking, wakefulness, and ear rubbing (Owais, Zawaideh & Al-Batayneh, 2010; Getaneh et al., 2018). Different combinations of these symptoms were reported among studies to an extent that a lacking consensus is evident, particularly in the association of the general symptoms such as diarrhea, fever, or vomiting to teething (Massignan et al., 2016). Nevertheless, the majority of the studies as well as the American Academy of Pediatric Dentistry (AAPD) agreed that gingival irritation and increased salivation are associated with teething (Massignan et al., 2016; American Academy of Pediatric Dentistry, 2018), particularly on the day of eruption and 1 day after the eruption (Ramos-Jorge et al., 2011)^,^ and that teething children should not display serious illness (American Academy of Pediatric Dentistry, 2018).

The role of parents in the teething process of their children is crucial since they naturally follow the development of their children and can detect changes in their behavior, mood, or health (Massignan et al., 2016). It is shown that parents diagnose “teething” symptoms to teething more than to palpable or visible tooth eruption (Wake, Hesketh & Allen, 1999). Several studies have investigated the knowledge of parents about teething and its associated signs and symptoms pointing out a misunderstanding of the whole process as well as false beliefs (Wake, Hesketh & Allen, 1999; Sarrell et al., 2005; Owais, Zawaideh & Al-Batayneh, 2010; Adimorah, Ubesie & Chinawa, 2011; Elbur et al., 2015; Kumar et al., 2016; Getaneh et al., 2018; More, Sankeshwari & Ankola, 2019). According to Sarrell et al. (2005), some parents even believed in the association between infant morbidity and teething.

The long period of the teething process can create difficulty for parents to differentiate teething symptoms from other coincidental mild infections such as gastrointestinal or respiratory symptoms (Owais, Zawaideh & Al-Batayneh, 2010). Consequently, some parents can easily misdiagnose childhood diseases as teething (Ashley, 2001). The reduction of maternal humoral immunity which occurs around the time of eruption of primary teeth can result in children being prone to systemic illnesses contributing to parental confusion with the developing symptoms (Sood & Sood, 2010; Kumar et al., 2016). Furthermore, the teething period coincides with the walking age of children and that is a time when children start exploring their environment and increase their risk of infection by introducing different objects to their mouths (Massignan et al., 2016).

In Saudi Arabia, Kumar et al. (2016) and Elbur et al. (2015) found that parents had gaps in their knowledge of teething in Jazan and Taif regions as well as inappropriate pain-relieving practices. However, they did not determine the parents who had good knowledge about teething. Furthermore, they did not determine the sociodemographic factors associated with good knowledge of parents about teething. Age of the parents, their residence, education level and occupation, the family income, the number of children, and order of the child in the family were all suggested previously to influence the knowledge level of parents about teething (Owais, Zawaideh & Al-Batayneh, 2010; Kakatkar et al., 2012; El-Gilany & Abusaad, 2017). Moreover, the association between knowledge of the parents about teething and adopting proper pain-relieving practices was never addressed previously since good knowledge of the parents about teething may not necessarily translate into applying proper practices to deal with teething problems. Therefore, the present study aims to firstly assess the knowledge level and practices of Saudi parents regarding teething and then explore associated sociodemographic variables with good knowledge of teething to determine the relation between parents’ knowledge of teething and their adoption of pain-relieving practices.

## Methods

### Procedure

Ethical approval was obtained from the Ethical Committee of College of Dentistry, Qassim University (Reference Number: EA/m-2019-3010) before the current study was conducted. Furthermore, all participants in the present study were informed of their rights including their right to withdraw from the study at any time, the aim of the study, the procedures involved, and the benefits of their participation. The confidentiality of personal identification and demographic data was assured so that participation was entirely voluntary.

### Participants

This was a cross-sectional study conducted on a convenience sample of Saudi parents who were contacted through the four social networking sites Facebook, Twitter, Instagram, and WhatsApp. The inclusion criteria for the study were: parents who were residing in Saudi Arabia as well as speak, read, and understand the Arabic language, parents who had at least one healthy, with no chronic illness or congenital anomaly, preschool child in the age range of 6 months-3 years, parents who gave written consent to participate in the study, and parents who had an account on the social networking sites Facebook, Twitter, Instagram, or WhatsApp.

### Study measures

A structured electronic questionnaire in the Arabic language was shared with the parents through accounts of the authors from September 2019 to April 2020. The Google Forms platform was used because it is available with no requirement for the parents to register. The questionnaire which was adopted by Owais, Zawaideh & Al-Batayneh (2010) and which had been tested for validity and reliability in the Arabic language was used (Owais, Zawaideh & Al-Batayneh, 2010). The questionnaire consisted of three parts and aimed to assess: (1) the sociodemographic profile of the parents and their children (gender of the parent, age group of the parent (18–27 years/28–37 years), education level of the parent (never been to school/primary level/intermediate level/secondary level/university), occupation of the parent (education sector/health sector/other than the education or health sector/unemployed), the family monthly income (<1,680 USD/1,680–3,200 USD/>3,200 USD), and number of children in the family (1–3/4–6/>6) (six questions); in this part, we slightly modified few answer categories of the questions about age group of the parent, the education level, the family monthly income, and the number of children in the family, to meet what is common in the Saudi community, (2) the general knowledge and beliefs of parents regarding their children’s teething including the teething period (initiation and completion age), the first primary teeth to erupt, the relation of delayed eruption to systemic diseases and signs and symptoms of teething (13 questions), and (3) the practices that the parents would do to manage teething problems and relieve pain or discomfort including allowing the child to bite on a chilled object, allowing bottle feeding or nursing at night, use of systemic analgesics or topical analgesics to rub the gum, giving the child fluids to prevent dehydration and consulting the doctor (six questions). Answer categories for part two and three questions were agree/disagree/do not know.

At the beginning of the questionnaire, a clear statement was written that only parents who had healthy children (at least one) in the teething age range, who were able to understand Arabic, and who were residing in the country were the ones invited to participate and answer the study questionnaire. Furthermore, parents who were willing to participate in the study gave consent in the online questionnaire and were requested to enter their region of residence in the country.

### Data analysis

Statistical analysis of the data was conducted using the SPSS software, version 22.0 (SPSS Inc., Chicago, IL, USA). Percentages (± frequency) of parents’ responses to the questions which assessed their sociodemographic profile, their general knowledge regarding their children’s teething and its associated signs and symptoms, and the practices that the parents would do to manage teething problems and relieve the pain or discomfort were produced. Parental knowledge about teething was assessed by giving the parents a score (out of 13) based on their answers to the questions about their knowledge of teething and its associated signs and symptoms (part two). Every correct answer equaled one point. Parents who answered correctly ≥9 questions (≥70% of the questions in part 2) were classified as having good knowledge. Chi-square test (univariate approach) was then used to detect any significant difference in the association of parental knowledge about teething with the sociodemographic characteristics including parent’s gender, age group, education level and occupation, the family monthly income, as well as the number of children in the family. Binary logistic regression analysis (multivariate approach) was further used to ascertain the association of parents’ sociodemographic profiles with their knowledge of teething. With regards to parental adopted practices to manage teething problems, and to be able to assess the relationship between parental knowledge of teething and their adoption of pain-relieving practices, a second score was calculated (out of 6) to assess the appropriateness of the adopted practices based on parental responses to part three questions. The relationship between parental knowledge of the teething score and having appropriate practices score was determined using Spearman rank-order correlation coefficient analysis. A *p*-value of <0.05 was considered to be statistically significant.

## Results

### The sociodemographic profile of parents

A total of 1,499 parents responded and returned completed questionnaires. The returned questionnaires covered the five geographic regions of Saudi Arabia in the following proportions: central region (50%), eastern region (20%), western region (10%), northern region (15%), and southern region (5%). About 57.20% of the parents were mothers or in the age range between 28–37 years. More than half of the parents (58.10%) held a university degree while only 1.10% of them had never been to school. The majority of parents (73.20%) were employed, and more than half of them (57.60%) earned a high monthly income (>3,200 USD). The great majority of the parents (75.30%) had four or more children.

### Parents’ knowledge of teething and its signs and symptoms

**Figure 1:**
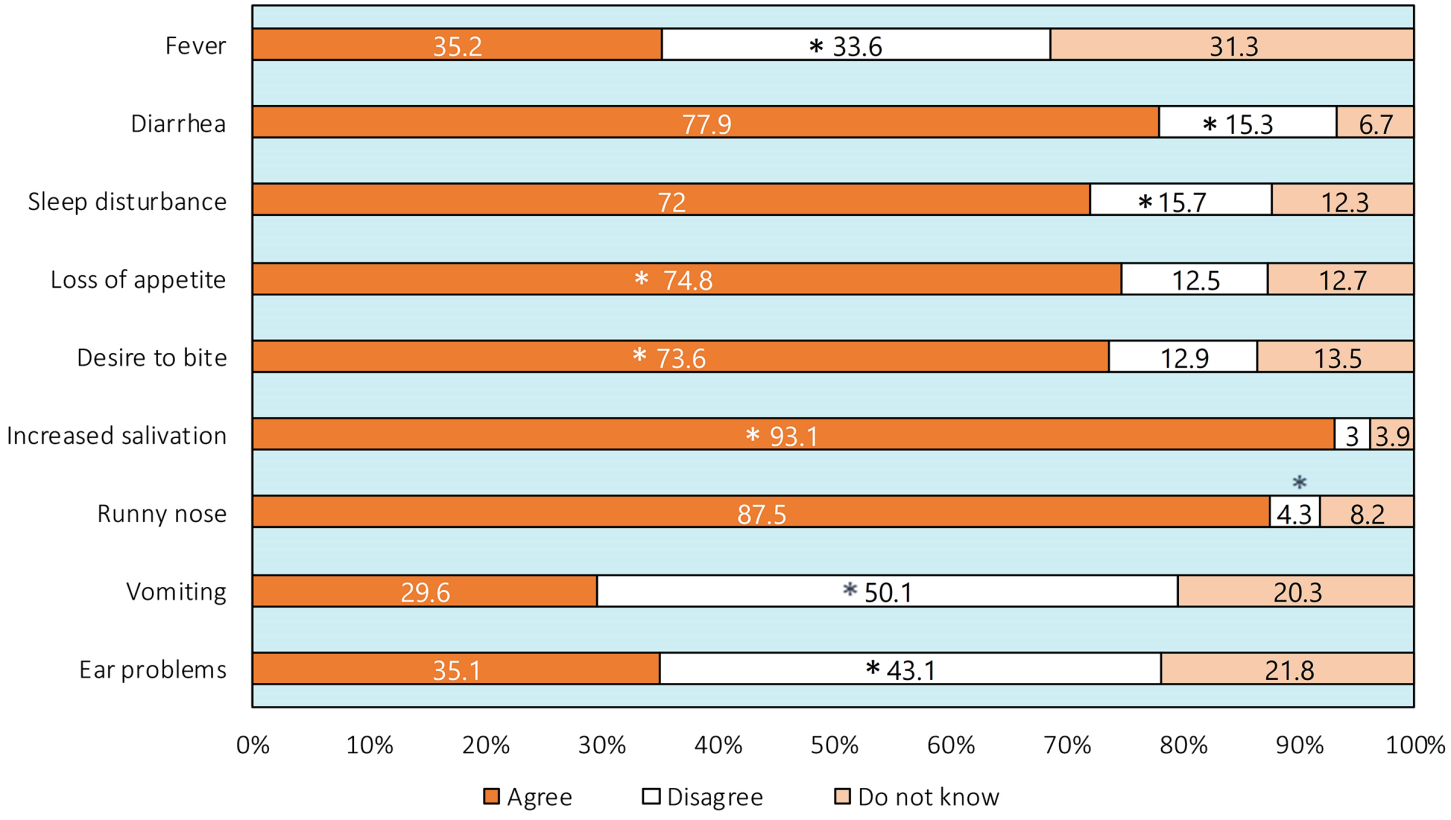
Parental responses in the questions about signs and symptoms of teething. An asterisk (*) indicates correct response.

Table 1 and Fig. 1 show the parents’ responses to the questions which assessed their knowledge about teething and its associated signs and symptoms. The mean knowledge score of the parents in that section was 6.43 ± 1.79 (out of 13). The mean knowledge score of mothers was 6.49 ± 1.75 while it was 6.34 ± 1.84 for fathers. None of the parents answered all of the questions correctly. Moreover, only 168 parents (11.20%) correctly answered ≥9 questions out of 13. Less than half of the parents knew the age of starting tooth eruption, and only 5.1% of the parents knew the age of completion (Table 1). Furthermore, the majority of the parents (>70%) attributed runny nose, diarrhea, and sleep disturbance to teething. Just around a third of them believed that fever is not related to teething (Fig. 1).

**Table 1:** Frequency (%) of parental responses in the questions about teething beliefs.

	Response	N (%)
Baby teeth start to erupt around 6–7 months of age	*Agree*	621 (41.40)
	Disagree	553 (36.90)
	Do not know	325 (21.70)
The first teeth to appear in the mouth are the lower central incisors	*Agree*	1214 (81.00)
	Disagree	129 (8.60)
	Do not know	156 (10.40)
The eruption of teeth is complete at approximately 2 years of age	Agree	1347 (89.90)
	*Disagree*	76 (5.10)
	Do not know	76 (5.10)
Delayed eruption of teeth may be an indication for the presence of systemic disease	*Agree*	1023 (68.20)
	Disagree	193 (12.90)
	Do not know	283 (18.90)

**Note:**

Correct response is in italics.

### Parents’ adopted pain-relieving practices

The adopted pain-relieving practices by parents are shown in Table 2. The mean score of the parents was 3.01 ± 1.00 (out of 6). None of the parents chose the appropriate practice in all of the questions and only a third of them (32.60%) chose the appropriate practice in 4–5 questions out of 6. Less than half of the parents (40.40%) allowed their child to bite on a chilled object, while less than one quarter of them would not apply a topical analgesic to rub the gum (20.80%) and thought that nocturnal feeding is not proper practice (14.40%).

**Table 2:** Frequency (%) of parental responses toward the practices to relieve pain associated with teething.

	Response	N (%)
Allow the child to bite on a chilled object	*Agree*	605 (40.40)
	Disagree	499 (33.30)
	Do not know	395 (26.40)
Allow bottle feeding or nursing at night	Agree	1088 (72.60)
	*Disagree*	216 (14.40)
	Do not know	195 (13.00)
Use systemic analgesics	*Agree*	1109 (74.00)
	Disagree	214 (14.00)
	Do not know	180 (12.00)
Apply topical analgesics to rub the gums	Agree	1086 (72.40)
	*Disagree*	312 (20.80)
	Do not know	101 (6.70)
Give the child fluids to prevent dehydration	*Agree*	1041 (69.40)
	Disagree	302 (20.10)
	Do not know	156 (10.40)
Consult the doctor	*Agree*	1245 (83.10)
	Disagree	96 (6.40)
	Do not know	158 (10.50)

**Note:**

Correct response is in italics.

### Associated sociodemographic factors with good knowledge about teething

The sociodemographic profile of the parents and the percentage of parents with good knowledge of teething as well as the sociodemographic factors which were associated with good knowledge of the parents according to the chi-square test are shown in Table 3. While Table 4 shows the results of binary logistic regression analysis. Only factors that were associated with good knowledge of the parents about teething according to the chi-square test were entered in the regression model. The results of the regression model revealed that two factors were associated with good knowledge about teething; namely, the education level and the family monthly income (*p* < 0.05). Compared to parents who held a university degree, parents who had never been to school but were able to read and understand the Arabic language as well as those with intermediate and secondary education levels were less likely to have good knowledge of teething. On the contrary, parents who earned intermediate monthly income were more likely to have good knowledge about teething than those who earned high monthly income (Table 4).

**Table 3:** The sociodemographic profile of the parents and the variables associated with good knowledge of parents about teething according to the chi-square test.

Variable	Category	(%)	N (%) with good knowledge	*p*-value
Parent’s gender	Male	42.80	66 (10.30)	0.505
	Female	57.20	98 (11.40)	
Parent’s age group	18–27	42.80	66 (10.30)	0.504
	28–37	57.20	98 (11.40)	
Parent’s education level	Never been to school	1.10	0 (0.00)	<0.0001*
	Primary	5.90	3 (3.40)	
	Intermediate	11.20	7 (4.20)	
	Secondary	23.70	29 (8.20)	
	University	58.10	125 (14.40)	
Family monthly income	<1,680 USD	27.60	29 (7.00)	<0.0001*
	1,681–3,200 USD	14.90	83 (37.20)	
	>3,200 USD	57.60	52 (6.00)	
Parent’s occupation	Education sector	20.30	34 (11.10)	0.904
	Health sector	25.80	43 (11.10)	
	Other	27.20	47 (11.50)	
	Unemployed	26.80	40 (10.00)	
Number of children in the family	1–3	24.70	26 (7.00)	0.02*
	4–6	39.80	71 (11.90)	
	>6	35.50	67 (12.60)	

**Note:**

* *p* < 0.05.

**Table 4:** The sociodemographic variables associated with good knowledge of parents about teething according to binary logistic regression analysis.

Variable	Category	B	OR	95% CI	*p*-value
Parent’s education level	Never been to school	−1.20	0.29	[0.12–0.73]	0.008*
	Primary	−0.12	0.88	[0.59–1.33]	0.562
	Intermediate	−0.58	0.55	[0.40–0.76]	<0.001*
	Secondary	−0.24	0.78	[0.61–0.98]	0.036*
	University^Reference^				
Family monthly income	<1,680 USD	0.05	1.06	[0.84–1.33]	0.621
	1,681–3,200 USD	1.89	6.63	[4.95–8.89]	<0.001*
	>3,200 USD^Reference^				
Number of children in the family	1–3	0.17	1.19	[0.71–2.00]	0.505
	4–6	−0.13	0.87	[0.59–1.28]	0.481
	>6^Reference^				

**Notes:**

* *p* < 0.05.

CI, confidence interval; OR, odds ratio.

### Correlation between parents’ knowledge of teething and their pain-relieving practices

**Figure 2:**
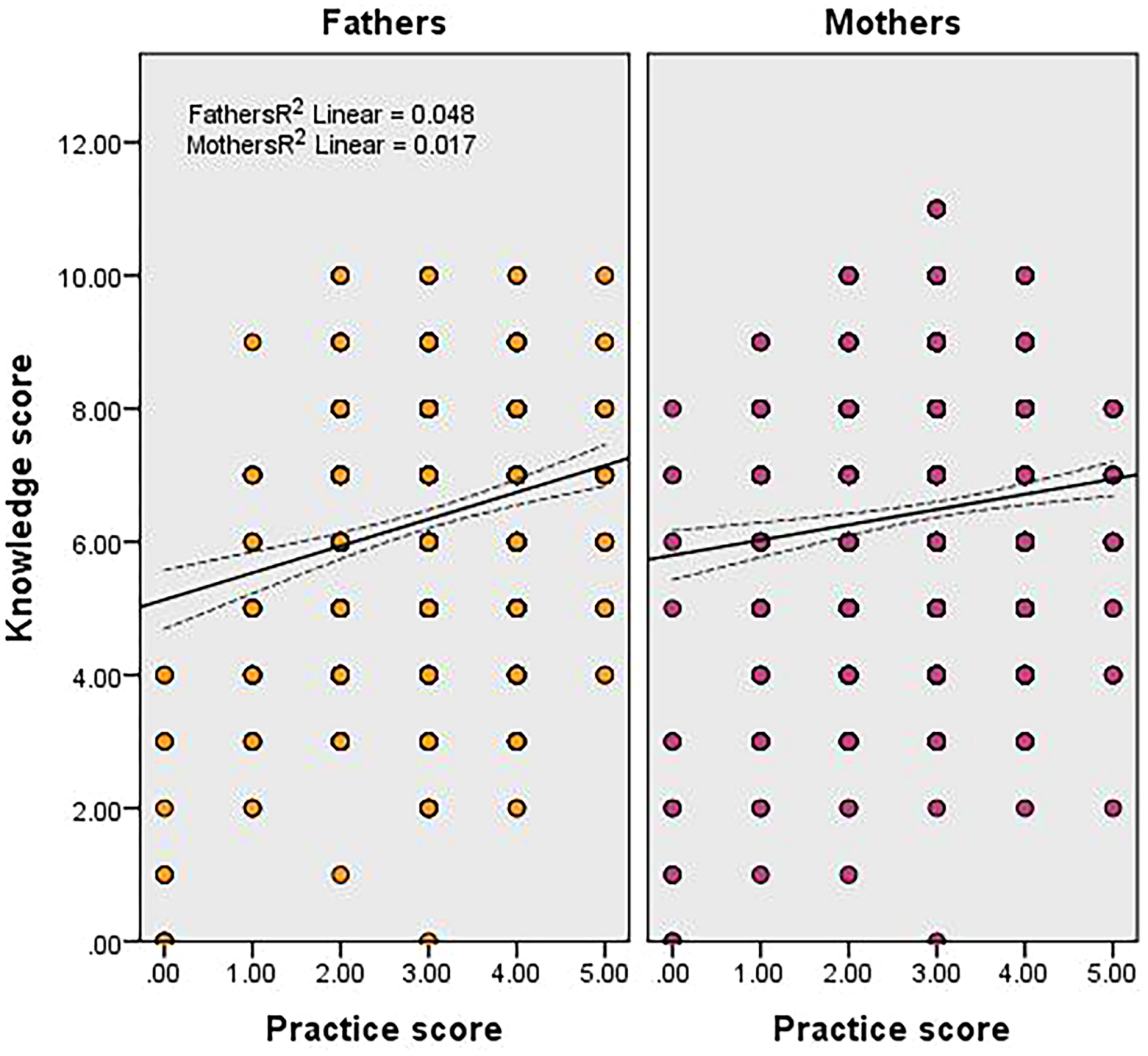
Relationship between the knowledge score and the practice score of fathers and mothers. The middle lines show the line of best fit for the correlation and the two lateral lines show the 95% confidence intervals for the mean values.

There was a weak and significant positive correlation between the knowledge score of parents about teething and their pain-relieving practices score regardless of their gender (fathers: *r* = 0.220, mothers: *r* = 0.132, *p* < 0.0001) (Fig. 2).

## Discussion

Parental knowledge about teething is a topic that has been discussed in the context of several populations, including Saudi Arabia (Owais, Zawaideh & Al-Batayneh, 2010; Adimorah, Ubesie & Chinawa, 2011; Kakatkar et al., 2012; Elbur et al., 2015; Kumar et al., 2016; Getaneh et al., 2018; El-Gilany & Abusaad, 2017; More, Sankeshwari & Ankola, 2019). However, few studies have determined the percentage of parents who have good knowledge about teething using a scoring system, as well as the sociodemographic factors associated with good knowledge about teething (Owais, Zawaideh & Al-Batayneh, 2010; El-Gilany & Abusaad, 2017). Furthermore, none have assessed the correlation between parental knowledge of teething and their adoption of pain-relieving practices. The current study is the first to address all of these points together using a large sample of parents from all regions of Saudi Arabia. The goal is to provide an in-depth investigation of the knowledge and practices of the parents, the factors associated with good knowledge, and whether or not good knowledge is translated into proper pain-relieving practices.

Our findings demonstrate that 11.2% of parents showed good knowledge about teething, which is close to the result reported in Egypt (16.8%) (El-Gilany & Abusaad, 2017), but lower than that reported in Jordan (38.5%) (Owais, Zawaideh & Al-Batayneh, 2010). The questions with the poorest performance concerned those about the teething period and the association of runny nose, diarrhea, sleep disturbance, fever, and ear problems with teething. The top four signs and symptoms reported by parents to be associated with teething included increased salivation (93.1%), runny nose (87.5%), diarrhea (77.9%), and loss of appetite (74.8%). Increased salivation and diarrhea were reported by the great majority of parents in Jazan and Taif regions of Saudi Arabia as teething symptoms (Elbur et al., 2015; Kumar et al., 2016). Additionally, Alghadeer et al. (2021) reported that 62% of Saudi mothers attribute childhood diarrhea to teething. Several studies report that parents believe that diarrhea is one of the most common teething symptoms (Barlow, Kanellis & Alayton, 2002; Sarrell et al., 2005; Adimorah, Ubesie & Chinawa, 2011; Kakatkar et al., 2012; Getaneh et al., 2018; More, Sankeshwari & Ankola, 2019; Yousif, 2020).

The percentage reported for runny nose in the current study (87.5%) is greater than that reported in previous studies (5.7–46%) (Baykan et al., 2004; Owais, Zawaideh & Al-Batayneh, 2010; Kakatkar et al., 2012; Elbur et al., 2015; Kumar et al., 2016; El-Gilany & Abusaad, 2017; Yousif, 2020). In this context, prospective studies on signs and symptoms of teething children have reported conflicting results in the association of diarrhea, fever, runny nose, and sleep disturbance with teething. Macknin et al. (2000) and Memarpour, Soltanimehr & Eskandarian (2015) could not find an association between diarrhea and fever with teething. Additionally, Macknin et al. (2000) could not find an association with sleep disturbance, while Memarpour, Soltanimehr & Eskandarian (2015) did find an association. In contrast, Ramos-Jorge et al. (2011) found an association between diarrhea, runny nose, sleep disturbance, and teething but did not find an association with fever. As such, there is no evidence to suggest that such general signs and symptoms are specific to teething and allow a diagnosis to be made confidently for a child without ruling out other possibilities (Oziegbe et al., 2011). Several studies have reported these as teething myths among parents and even health professionals (Owais, Zawaideh & Al-Batayneh, 2010; Kakatkar et al., 2012; Ispas, Mahoney & Whyman, 2013; El-Gilany & Abusaad, 2017).

The fact that parents believe that their child’s diarrhea is due to teething is concerning as parents may think that teething diarrhea is not serious, they may think that it does not lead to dehydration, and consequently, they will not seek medical advice (Sarrell et al., 2005). Furthermore, Massignan et al. (2016) pointed out that teething could lead to a rise in temperature, but it is not characterized as fever. Maximal recorded readings of tympanic and axillary temperatures were 36.8 °C and 36.6 °C during teething (Ramos-Jorge et al., 2011).

Children are considered feverish when they have a temperature of 38.3 °C or greater (Long, 2016). However, whether parents were aware of the difference between fever and a mild rise in temperature is unclear. Parents likely consider any rise in temperature as fever. As noted in one study conducted in a Saudi region, parents had poor knowledge of determining the threshold for defining fever (Elbur, 2014). Runny nose, ear problems, and fever are considered symptoms of a concurrent respiratory problem (El-Gilany & Abusaad, 2017). Bearing in mind the current COVID-19 pandemic, parents must differentiate COVID-19 symptoms from teething symptoms.

Concerning adopting proper practices to deal with teething pain, the parents’ scores were not better than their knowledge scores. Just 40% would give their child a chilled object to bite on, while a majority of them (72%) would allow bottle feeding at night in an attempt to soothe their child’s pain, as well as apply a topical analgesic to rub the gums (72.4%). Kumar et al. (2016) reported that around half of Saudi mothers in the Jazan region allowed bottle feeding at night and applied a topical analgesic to soothe the pain.

Few studies considered applying a topical analgesic an appropriate practice (Owais, Zawaideh & Al-Batayneh, 2010; Kakatkar et al., 2012). Current regulations of the American Academy of Pediatrics (AAP) and the AAPD discourage it due to the potential harm of these products to infants and the risk of developing methemoglobinemia from benzocaine. The use of oral analgesics and chilled (not frozen) rings is encouraged instead (American Academy of Pediatric Dentistry, 2018). Alternatively, rubber teething rings, chewing on a cool washcloth, and gentle gum massage are considered safe and appropriate for symptomatic relief (Thompson & Huntington, 2019). Not only is there a potentially harmful effect of topical analgesics, they often wash out of the mouth quickly, rendering them ineffective (American Academy of Pediatrics, 2016). In the case of night bottle feeding, it has been associated with early childhood caries, which has been reported to be prevalent in the Saudi community (Al-Haj Ali et al., 2021).

In the present study, the knowledge scores of fathers and mothers correlated positively with the practice scores. This indicates that good knowledge of parents regardless of their gender was translated into proper practices and consequently points out no difference between Saudi mothers and fathers in proxy reporting about teething. Yet what is important to remember is the low percentage of parents who had good knowledge about teething. Feldens et al. (2010) reported that dentists had little influence on the management of teething symptoms. This perhaps means that the concept of establishing an early dental home is not practiced in Saudi Arabia as providing anticipatory guidance regarding teething should be an important component of the first dental visit (American Academy of Pediatric Dentistry, 2018). Consequently, other health professionals like medical doctors, pediatricians, and gynecologists, particularly those serving the general population and working within the ministry-of-health facilities, can utilize the opportunity to educate parents or expectant mothers about teething during antenatal checkups and immunization visits, which are scheduled regularly and are free of charge (Kakatkar et al., 2012, Al-Hatalani & Al-Haj Ali, 2019).

It has been reported that sociodemographic characteristics can affect caregivers’ perceptions regarding their child’s oral health and is related to oral health conditions (Al-Haj Ali & Alshabaan, 2020). These include a family’s socioeconomic status, which may be evaluated by income and education. In the present study, the education level of the parents and the families’ monthly incomes were associated with good knowledge among the parents about teething. Parents who had a university degree and those who earned intermediate monthly incomes were more likely to have good knowledge. Several authors state that higher education levels and incomes were associated with better knowledge among parents about teething (Owais, Zawaideh & Al-Batayneh, 2010; Kakatkar et al., 2012; El-Gilany & Abusaad, 2017). Feldens et al. (2010) also reported that the risk of reporting teething symptoms was higher for children from families with higher income. This was attributed to a likelihood of families with higher socioeconomic status having positive health attitudes that include paying more attention to symptoms that appear early in life (Feldens et al., 2010; Kakatkar et al., 2012).

The strengths of the current study include the use of a reliable questionnaire for the survey. Furthermore, we have targeted a large sample size from all Saudi regions. Nevertheless, the present study still has some limitations that warrant caution in the interpretation of the results. First, we have used a convenience sample of parents. Hence, we do not claim our study to be representative or to provide an accurate reflection of knowledge levels, despite that different sociodemographic groups were included in the studied population. Nevertheless, the present study still provides baseline data for future studies. The second limitation is that this was a cross-sectional study, and we cannot infer causation from any of the associations that we observed. Another limitation was that the parents were questioned retrospectively about teething, which may have caused recall bias, as well as not addressing the order of the child in the family. However, all parents had a child in the teething period (up to 3 years of age), so forgetting this period is unlikely.

## Conclusion

A very low percentage of Saudi parents—mainly those with the highest education level and intermediate monthly income—had good knowledge of teething, which translated into appropriate practices to soothe the child’s pain regardless of the parent’s gender. Saudi parents and expectant mothers should receive anticipatory guidance related to teething from health professionals, including medical doctors, pediatricians, and gynecologists. Improving knowledge and practices of parents about teething and the importance of seeking medical checkup to rule out other problems when general symptoms such as diarrhea, runny nose, sleep disturbance, or fever in children develop should result in an uneventful teething period. Furthermore, it could help with timely diagnosis and immediate management of childhood diseases, which should reduce morbidity and the risk of complications among children.

## Supplemental Information

10.7717/peerj.13948/supp-1 Supplemental Information 1 The questionnaire used in the study.

10.7717/peerj.13948/supp-2 Supplemental Information 2 Data: SPSS file.

## Additional Information and Declarations

### Data Availability

The following information was supplied regarding data availability: The raw data is available in the Supplemental File.
