# Supplementary material for: Levels and correlates of knowledge of teething among Saudi Arabian families
Source: PeerJ. 2022 Aug 18;10:e13948. doi: 10.7717/peerj.13948 (PMC9393006; doi:10.7717/peerj.13948)
Supplement: Supplemental Information 1 [file peerj-10-13948-s001.docx]

**Part 1:**

1. **Gender**

- **Male**
- **Female**

1. **Age**

- 18-27 years.
- 28-37 years.
- 38-47 years.
- >47 years.

1. **Occupation**

- Education sector.
- Health sector.
- Other.
- Unemployed

1. **Education level**

- Never been to school.
- Primary.
- Secondary.
- Intermediate.
- University.

1. **Family monthly income**

- <1680 USD
- 1680 to 3200 USD
- >3200 USD

1. **Number of children**

- 1-3 children.
- 4-6 children.
- 6 children.

Part 2:

1. **‘Baby teeth’ start to erupt around 6–7 months of age:**

- Agree
- Disagree
- Don’t know

1. **The first teeth to appear in the mouth are the lower central incisors:**

- Agree
- Disagree
- Don’t know

1. **The eruption of teeth is complete at approximately 2 years of age:**

- Agree
- Disagree
- Don’t know

1. **Delayed eruption of teeth may be an indication for the presence of systemic disease:**

- Agree
- Disagree
- Don’t know

1. **Signs and symptoms caused by teething include:**

- Fever. (Agree Disagree Don’t know)
- Diarrhea. (Agree Disagree Don’t know)
- Sleep disturbance. (Agree Disagree Don’t know)
- Loss of appetite. (Agree Disagree Don’t know)
- Desire to bite. (Agree Disagree Don’t know)
- Increased salivation. (Agree Disagree Don’t know)
- Runny nose. (Agree Disagree Don’t know)
- Vomiting. (Agree Disagree Don’t know)
- Ear problems. (Agree Disagree Don’t know)

Part 3:

1. To relieve pain associated with teething:

- Allow the child to bite on a chilled object. (Agree Disagree Don’t know)
- Allow bottle feeding or nursing at night. (Agree Disagree Don’t know)
- Use systemic analgesics. (Agree Disagree Don’t know)
- Apply topical analgesics to rub the gums. (Agree Disagree Don’t know)
- Give the child fluids to prevent dehydration. (Agree Disagree Don’t know)
- Consult the doctor. (Agree Disagree Don’t know)
